# Roles of Nrf2/HO-1 and ICAM-1 in the Protective Effect of Nano-Curcumin against Copper-Induced Lung Injury

**DOI:** 10.3390/ijms241813975

**Published:** 2023-09-12

**Authors:** Wedad S. Sarawi, Ahlam M. Alhusaini, Hanan K. Alghibiwi, Juman S. Alsaab, Iman H. Hasan

**Affiliations:** Department of Pharmacology and Toxicology, College of Pharmacy, King Saud University, P.O. Box 22452, Riyadh 11495, Saudi Arabia; aelhusaini@ksu.edu.sa (A.M.A.); halghibiwi@ksu.edu.sa (H.K.A.); 443203447@student.ksu.edu.sa (J.S.A.); ihasan@ksu.edu.sa (I.H.H.)

**Keywords:** curcumin, lung toxicity, inflammation, copper sulfate, oxidative stress, Nrf2/HO-1 pathway, ICAM-1

## Abstract

Copper (Cu) is an essential trace element for maintaining normal homeostasis in living organisms. Yet, an elevated level of Cu beyond homeostatic capacity may lead to oxidative damage of cellular components in several organs, including the lungs. This work investigated the effects of curcumin (Curc) and nano-curcumin (nCurc) against Cu-induced lung injury, accenting the roles of oxidative stress, inflammation, and the nuclear factor erythroid 2-related factor/heme oxygenase-1 Nrf2/HO-1 pathway. Rats were challenged with 100 mg/kg of copper sulfate (CuSO_4_) while being treated with Curc or nCurc for 7 days. Cu-triggered lung oxidative stress detected as dysregulation of oxidative/antioxidant markers, a downregulation of Nrf-2/HO-1 signaling, and an increase in the inflammatory markers interleukin-6 (IL-6), tumor necrosis factor-alpha (TNF-α), and intracellular adhesion molecule-1 (ICAM-1). Additionally, it decreased the expression of lung-specific proteins, surfactant protein-C (SP-C), and mucin-1 (MUC-1), induced apoptosis, and caused changes in lung histology. Curc and nCurc alleviated CuSO_4_-induced lung injury by suppressing oxidative damage and inflammation and activating Nrf-2/HO-1. They also prevented apoptosis and restored the normal expression of SP-C and MUC-1. We concluded that nCurc exhibited superior efficacy compared with Curc in mitigating CuSO_4_-induced lung injury. This was associated with reduced oxidative stress, inflammation, and apoptotic responses and increased Nrf2/HO-1 signaling and expression of SP-C and MUC-1.

## 1. Introduction

Copper (Cu) is a crucial trace element and a cofactor of several redox enzymes. It is involved in numerous biological functions, including blood coagulation, neurotransmitter synthesis, antioxidant defense, energy production, and cellular metabolism [1,2]. The maintenance of Cu homeostasis is tightly regulated via the balancing of its absorption, excretion, and circulating levels. Cu possesses the potential for toxicity owing to its chemical redox potential and capacity to engage in free radical reactions [3]. It is widely used in many industries to synthesize electronics, building materials, wood protection, and pesticides. Cu toxicity may result from acute or chronic exposure to excess Cu due to accidents, occupational hazards, and environmental pollution. It can also be accumulated due to genetic defects, as in the case of Wilson’s disease, an inherited mutation in the *ATP7B* gene that encodes for a protein responsible for Cu excretion [4,5,6]. Such toxicity increases the risk of developing neurological, hepatic, and renal diseases, which are attributed primarily to oxidative stress, DNA damage, and cell apoptosis [4,7,8].

Copper sulfate (CuSO_4_) is an inorganic compound frequently used in agriculture, analytical, and tissue culture laboratories as it possesses pesticidal, redox potential, and antimicrobial actions, respectively. However, accidental or deliberate intoxication with Cu may cause multiorgan toxicity, which can be life-threatening [9,10]. The acute toxicity of CuSO_4_ can cause hepatitis, jaundice, intravascular hemolysis, methemoglobinemia, erosive gastritis, acute tubular necrosis, and rhabdomyolysis [11,12]. The toxic effect of CuSO_4_ ingestion on the lungs has been reported previously as it exhibits corrosive effects on mucous membranes [13]. Repeated exposure to CuSO_4_ pentahydrate via inhalation results in a dose-related pulmonary inflammatory response and increased lung weight due to epithelial hyperplasia in rats [14]. Chelating therapies, including D-penicillamine, trientine, and deferoxamine (DFO), are still used to control Cu toxicity, just like with other metals [15]. However, the adoption of safer substitutes is required owing to the partial efficacy and side effects of these agents.

Since the lungs are usually exposed to substantial oxygen levels, they possess a collection of enzymatic and non-enzymatic antioxidants that often function extensively to counter any potential oxidative assaults [16]. It was recently reported that an elevation in urinary Cu levels was directly correlated with a higher risk of lung fibrotic changes [17]. In addition, there is an association between environmental Cu exposure and the risk of developing lung cancer [18]. Copper oxide nanoparticles (CuO NPs) induce lung epithelial cell death and pulmonary fibrosis in mice [19]. Likewise, studies have revealed that Cu has the capacity to elicit oxidative stress and provoke an accumulation of reactive oxygen species (ROS) [20,21]. ROS incur cycles of catastrophic events that initiate several inflammatory responses and cytokine release [22,23]. Persistent release of these mediators can cause tissue injury by activating the nuclear factor kappa B (NF-κB) pathway, mitogen-activated protein kinases (MAPKs), and apoptosis [4,24].

Curcumin (Cur) is a natural phytochemical present in turmeric with substantial antioxidant, anti-inflammatory, immunomodulatory, antibacterial, and antiviral actions [25,26,27]. Curc exerts in vivo and in vitro antioxidant effects via multiple mechanisms as it neutralizes ROS and reactive nitrogen species (RNS) [28,29]. This neutralization is attributed to the abundance of conjugated double bonds in its structure which serve as efficient electron donors in the counteraction of reactive species in many redox reactions [30]. Curc has some therapeutic effects in acute and chronic lung diseases like acute lung injury (ALI), asthma, pneumonia, and chronic obstructive pulmonary disease (COPD) [31,32]. While there are existing studies that demonstrate its protective efficacy against viral-induced lung injury in acute respiratory distress syndrome in mice [33] and ventilator-induced lung injury (VILI) in rats [34], and reduced symptoms, hospital stay, and mortality in COVID-19 patients [35], the protective effect of Curc against lung injury caused by CuSO_4_ has not yet been studied. Some of the therapeutic potential of Curc is hindered by its inadequate solubility in water, limited absorption and systemic bioavailability, fast metabolism, physicochemical instability, and low effectiveness in penetrating and targeting specific sites [36]. Therefore, this study investigated the impact of CuSO_4_-induced lung injury in rats and explored the potential protective effects of Curc and its nanoformulation (nano-curcumin, nCurc) against such injury. These were achieved by assessing the pulmonary expression of the nuclear factor erythroid 2-related factor 2 (Nrf2)/heme oxygenase-1 (HO-1) signaling pathway, inflammatory cytokines, and intracellular adhesion molecule-1 (ICAM-1) expression in rats.

## 2. Results

### 2.1. Curc and nCurc Mitigate Oxidative Stress after CuSO_4_-Induced Lung Injury

**Figure 1:**
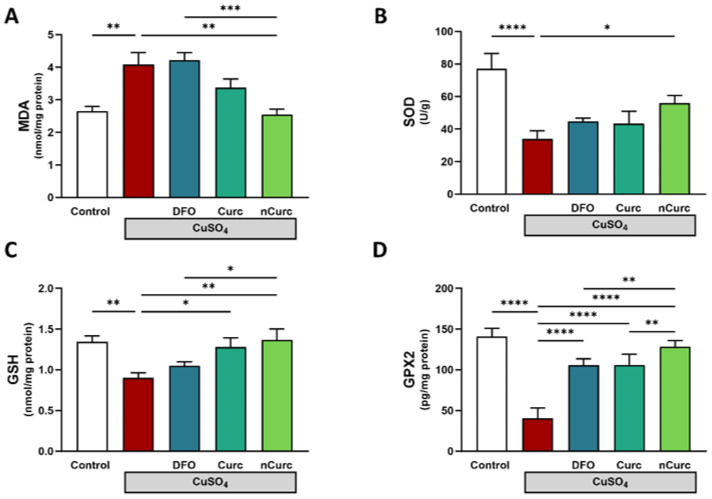
Curc and nCurc mitigate oxidative stress after CuSO_4_-induced lung injury. Treatment with nCurc decreased (**A**) MDA and increased (**B**) SOD activity, (**C**) GSH, and (**D**) GPX2. Curc increased GSH and GPX2 after CuSO_4_ overexposure. Data are depicted as mean ± SEM (*n* = 7). * *p* ≤ 0.05, ** *p* ≤ 0.01, *** *p* ≤ 0.001, and **** *p* ≤ 0.0001.

Lipid peroxidation, indicated by MDA, was significantly increased in the lung tissue homogenate of CuSO_4_-intoxicated rats relative to that of the control rats (*p* ≤ 0.01; Figure 1A). In contrast, the SOD activity (*p* ≤ 0.0001; Figure 1B), GSH level (*p* ≤ 0.01; Figure 1C), and GPX2 level (*p* ≤ 0.0001; Figure 1D) were reduced after such injury in comparison to those of the control group. The concurrent use of nCurc showed profound mitigation of pulmonary oxidative stress by lowering the MDA level and increasing SOD, GSH, and GPX2 relative to the CuSO_4_ group (*p* ≤ 0.01, *p* ≤ 0.05, *p* ≤ 0.01, and *p* ≤ 0.0001, respectively). Curc exhibited significant elevations in GSH and GPX-2 (*p* ≤ 0.05, *p* ≤ 0.0001), while DFO increased GPX2 (*p* ≤ 0.0001) only following CuSO_4_ overexposure.

### 2.2. Curc and nCurc Attenuate CuSO_4_-Induced Histopathological Changes in Lung Tissues

**Figure 2:**
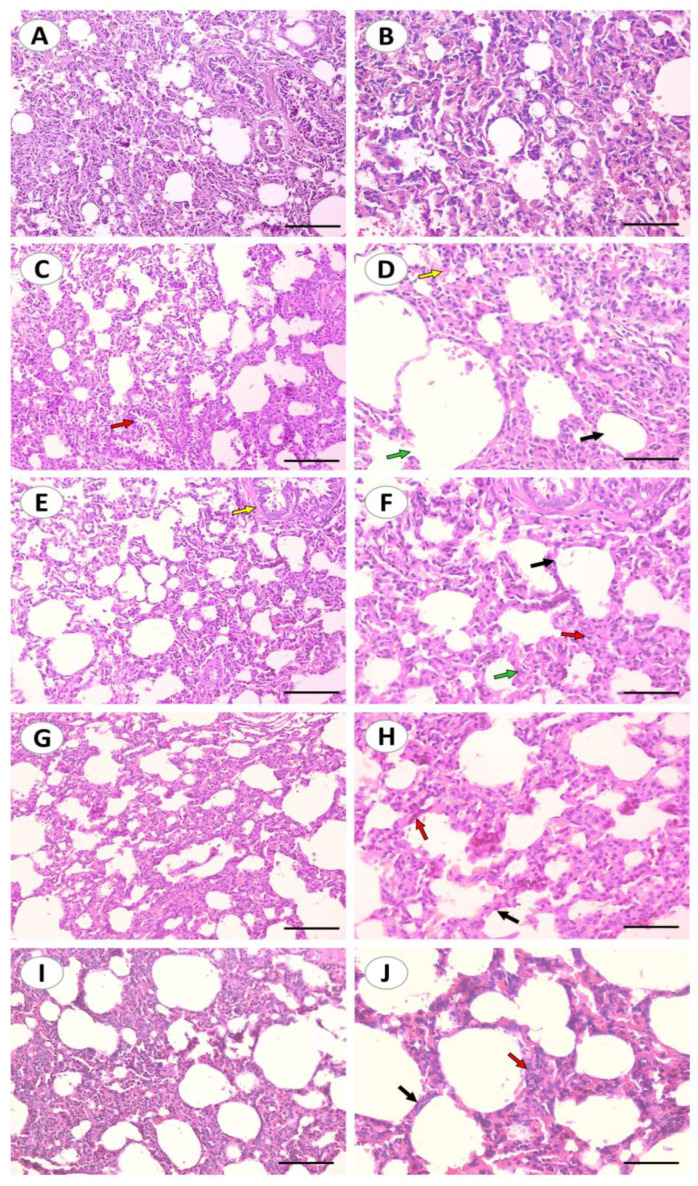
Curc and nCurc improve tissue appearance and reverse CuSO_4_-induced lung injury. Representative images of H&E-stained lung sections of control rats (**A**,**B**) showing normal lung histology. CuSO_4_-challenged rats (**C**,**D**) showing inter-alveolar septum (black arrow), intra-alveolar hemorrhage and congestion (yellow arrow), bronchus (red arrow), ruptured alveolus (green arrow). Cu-challenged rats treated with DFO (**E**,**F**) showing alveoli with thin inter-alveolar septum (black arrow), mild infiltration of lymphocytes (red arrow), bronchus (yellow arrow), type I and type II pneumocytes are also seen (green arrow). CuSO_4_-challenged rats treated with Curc (**G**,**H**) and nCurc (**I**,**J**) showing alveoli with normal inter-alveolar septum (black arrow), type I and type II pneumocytes are also seen (red arrow). (200×: (**A**,**C**,**E**,**G**,**I**), scale bar = 200 µm, 400×: (**B**,**D**,**F**,**H**,**J**), scale bar = 100 µm).

Histological staining was utilized to assess the pathological changes that occurred in the lungs after CuSO_4_ exposure and their response to treatments (Figure 2A–J). The control group showed a normal bronchus, alveoli with thin inter-alveolar septa, and type I and II pneumocytes. In contrast, CuSO_4_ induced some pathological changes which were identified as infiltration of lymphocytes, intra-alveolar hemorrhage, and ruptured alveoli. Treatment with DFO showed alveoli with thin inter-alveolar septa and mild lymphocyte infiltration. Curc and nCurc improved lung tissue appearance and reversed the pathological changes induced by CuSO_4_.

### 2.3. Curc and nCurc Modulate Pulmonary Keap-1/Nrf-2/HO-1 Signaling after CuSO_4_-Induced Lung Injury

**Figure 3:**
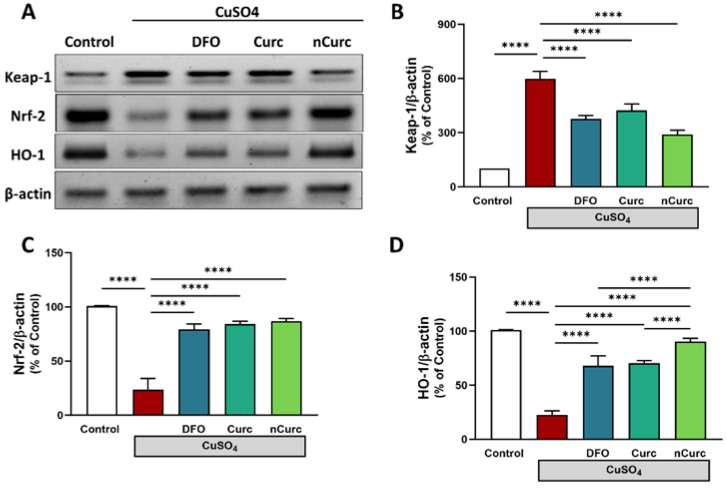
Curc and nCurc modulate pulmonary Keap-1/Nrf-2/HO-1 signaling after CuSO_4_-induced lung injury (**A**–**D**). Exposure to CuSO_4_ caused an increase in Keap-1 and a decrease in Nrf-2 and HO-1 protein expression. Treatment with DFO, Curc, and nCurc improved the expression of these proteins. Data are depicted as mean ± SEM (*n* = 7). **** *p* ≤ 0.0001.

The protein expression of pulmonary Keap1 was significantly upregulated, while Nrf-2 and HO-1 were significantly downregulated in CuSO_4_-intoxicated rats, as compared with the control group (*p* ≤ 0.0001, as shown in Figure 3). However, treatment of those rats with DFO, Curc, and nCurc exhibited a significant decrease in Keap-1 expression and an increase in Nrf-2 and HO-1 expression (*p* ≤ 0.0001). Notably, nCurc upregulated HO-1 expression significantly compared with DFO or Curc (*p* ≤ 0.0001).

### 2.4. Curc and nCurc Ameliorate Inflammation after CuSO_4_-Induced Lung Injury

**Figure 4:**
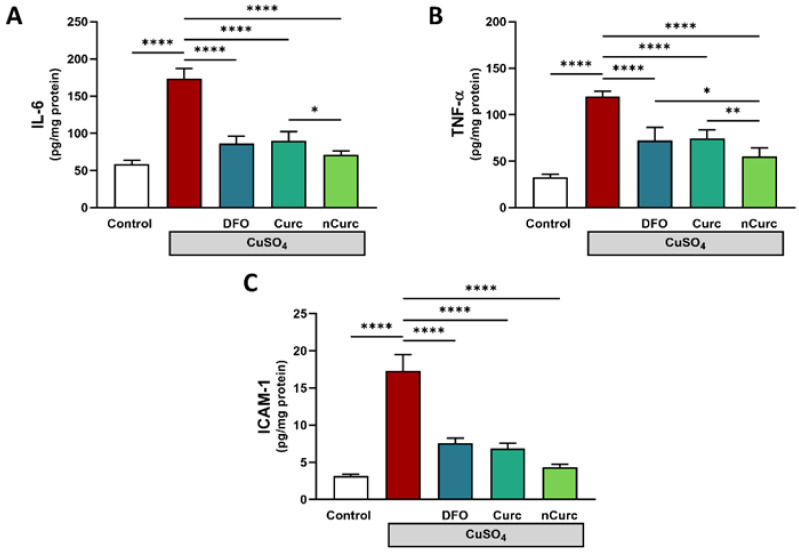
Curc and nCurc ameliorate pulmonary inflammation after CuSO_4_-induced lung injury (**A**–**C**). Cu exposure caused an increase in inflammatory marker levels. Treatment with DFO, Curc, and nCurc ameliorated the inflammation. Data are depicted as mean ± SEM (*n* = 7). * *p* ≤ 0.05, ** *p* ≤ 0.01, **** *p* ≤ 0.0001.

To confirm the lung-protective effects of DFO, Curc, and nCurc, we measured the levels of inflammatory biomarkers (IL-1β, TNF-α, and ICAM-1) in lung tissues. Rats exposed to CuSO_4_ demonstrated a significant increase in these markers (Figure 4) compared with the control group. Nevertheless, treatment with antioxidants effectively mitigated the levels of these inflammatory markers in rats administered CuSO_4_, as demonstrated in Figure 4.

### 2.5. Curc and nCurc Restore SP-C and MUC-1 Levels after CuSO_4_-Induced Lung Injury

**Figure 5:**
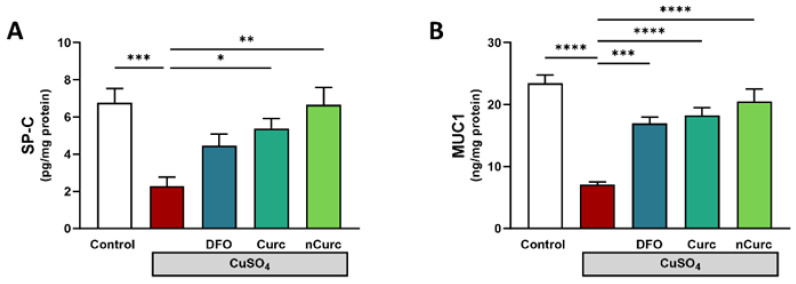
Curc and nCurc restore the pulmonary expression of SP-C and MUC-1 after CuSO_4_-induced lung injury (**A**,**B**). Data are depicted as mean ± S.E.M. (*n* = 7). * *p* ≤ 0.05, ** *p* ≤ 0.01, *** *p* ≤ 0.001, **** *p* ≤ 0.0001.

To further confirm the protective effects of Curc and nCurc, we measured the levels of SP-C and MUC-1 in the lung tissues. CuSO_4_-administered rats demonstrated a significant decrease in these markers (Figure 5) compared with the control group. However, treatment with antioxidants effectively restored the levels of these inflammatory markers in rats administered CuSO_4_.

### 2.6. Curc and nCurc Prevent Apoptosis by Regulating BAX and Bcl-2 Gene Expression Levels after CuSO_4_-Induced Lung Injury

**Figure 6:**
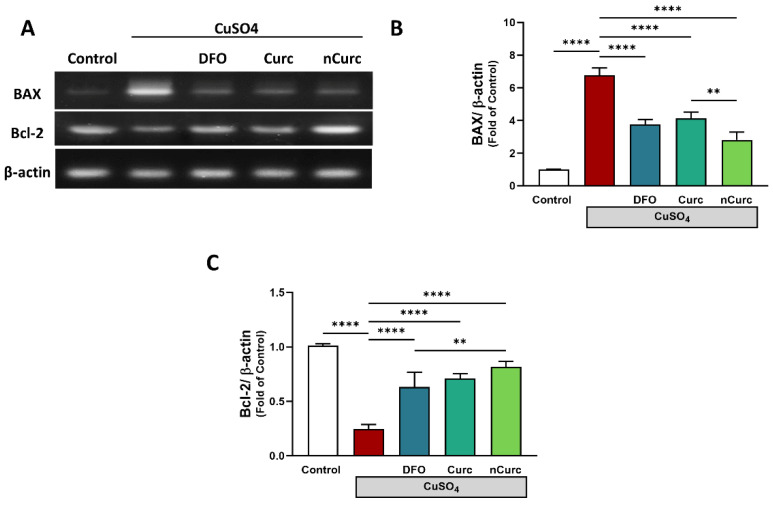
Curc and nCurc prevent apoptosis by regulating *BAX* and *Bcl-2* gene expression after CuSO_4_-induced lung injury (**A**–**C**). CuSO_4_ exposure caused an increase in *BAX* and a reduction in *Bcl2* mRNA levels. Treatment with DFO, Curc, and nCurc improved the levels of apoptotic markers. Data are depicted as mean ± SEM (*n* = 7). ** *p* ≤ 0.01, **** *p* ≤ 0.0001.

The CuSO_4_-administered group showed a significant upregulation of *BAX* gene expression (*p* ≤ 0.001) and downregulation of *Bcl-2* gene expression (*p* ≤ 0.001) compared with the control group, as depicted in Figure 6. Nevertheless, treatment with DFO, Curc, and nCurc significantly mitigated the effects of CuSO_4_ on gene expression (*p* ≤ 0.001) by restoring their average expression levels.

## 3. Discussion

Cu is a pivotal micromineral for many physiological processes in the body, such as cellular respiration, enzyme activation, immune responses, antioxidant defenses, and energy homeostasis [1]. Despite its importance, excess Cu can cause serious multiorgan toxicities if absorbed systemically through the lungs, skin, and gastrointestinal tract [9]. Extensive occupational exposure to Cu can cause severe lung diseases primarily from inhaled particulates from mining or metal fumes from smelting, welding, agriculture, or other related enterprises [5,37]. Cu accumulation is a risk for multiple tissue damage and fibrosis, including in the lungs [38]. Limited research has been conducted regarding pulmonary toxicity due to copper exposure. One case report revealed that the ingestion of CuSO_4_ caused severe pulmonary toxicity manifested by acute, bilateral pulmonary infiltrates and hypoxemia [13]. Yet, the deleterious effects and mechanistic consequences of this metal on the lungs are less addressed. Therefore, we investigated the role of Curc and its nanoform against CuSO_4_-induced pulmonary injury in rats, pointing towards the changes in inflammation, the Nrf2/HO-1 pathway, and the lung-functioning proteins SP-C and MUC-1.

The present study demonstrated that exposure to CuSO_4_ resulted in pulmonary oxidative stress, as evidenced by an elevation in lipid peroxidation levels and a reduction in SOD activity, GSH, and GPX2 levels. Cu has a redox catalytic reactivity that is crucial for many biological reactions. On the contrary, when its concentration surpasses a certain limit, this metal generates free radicals that are extremely reactive and toxic [39]. Cu can also alter the activity of electron respiratory chain proteins, which are crucial for releasing energy in the mitochondria, and produce excess ROS [40]. ROS are strong oxidizers that enhance lipid peroxidation, protein damage, DNA fragmentation, and cell death [4,41]. In addition, aberrations in the oxidant/antioxidant balance caused by Cu result in the oxidation of proteins’ sulfhydryl groups, thereby depleting GSH stores and reducing SOD activity in the lungs and other organs, as previously reported [42,43,44,45]. MDA is the most prominent byproduct of lipid peroxidation, and it crosslinks to tissue DNA or protein to form adducts, resulting in biomolecular damage [46]. GSH is a non-enzymatic antioxidant defense that acts directly on free radicals by quenching their reactivity, thus protecting cells from their destructive consequences, or indirectly by activating detoxification enzymes such as glutathione peroxidases, glutathione S-transferases, and glyoxalases [47,48,49]. SOD is an enzymatic antioxidant found in most organs, including the brain, liver, thyroid, and lungs. This enzyme converts toxic superoxide radicals to less reactive dioxygen and hydrogen peroxide [50,51]. Moreover, Cu causes downregulation of some antioxidant genes including *GPX2*, *GSR.*, and *KEAP1,* in A549 lung epithelial cells [48]. It competes with other metals inside the cells, displaces them from their metal binding sites, and further impairs cellular health and survival [52]. 

Considering the contribution of oxidative stress in the mechanism of Cu-induced lung injury, Curc can potentially mitigate such damage because of its antioxidant action and modulation of the Keap-1/Nrf-2/HO-1 signaling pathway. In the present study, rats that received Curc or nCurc concurrently with CuSO_4_ exhibited a notable reversal in pulmonary oxidative stress markers by decreasing MDA levels and increasing GSH, GPX2, and SOD activity. Previous studies reported that Curc possessed antioxidant effects against animal models of lung toxicity or injury induced by nicotine [53], elastase and cigarette smoke [54], bleomycin [55], cyclophosphamide [25], and even radiation [56]; it decreased MDA and enhanced GSH and antioxidant enzymes that parallel with our findings. The rats that received nCurc displayed more significant antioxidant effects than those observed in rats administered the native form of Curc. In the same context, the protective findings for Curc and nCurc were analyzed using histopathological observations of rat lungs after Cu injury. The histopathological examination of the lung tissue of rats exposed to CuSO_4_ demonstrated degenerative changes that supported the previous biochemical results. These changes included pulmonary congestion, intra-alveolar hemorrhage, ruptured alveoli, and lymphocyte infiltration. The antioxidant agents almost restored normal lung tissue architecture with no sign of ruptured alveoli or lymphocyte infiltration.

Keap-1/Nrf-2/HO-1 is a prominent pathway of cytoprotective responses to both endogenous and exogenous stresses resulting from ROS. An essential signaling protein within this pathway is the transcription factor Nrf2. The significance of Nrf2 in the pulmonary system has emerged via knockout experiments in which mice lacking the *Nrf2* gene exhibit increased susceptibility to various chemically induced pulmonary toxicities and pathologies [57]. The disruption of the *Nrf2* gene in mice results in prompt and severe emphysema upon exposure to cigarette smoke [58]. Moreover, Nrf2^−/−^ mice exhibited severe lung damage characterized by increased protein permeability, macrophage inflammation, and epithelial injury after hyperoxia exposure [59]. Previous studies have reported that Cu toxicity can cause downregulation of Nrf2 and HO-1 in the liver [60], brain [61,62], and testes [45], which supports our findings.

The beneficial effects of Curc and nCurc against CuSO_4_-induced oxidative lung injury might be attributed to the activation of Nrf2/HO-1 signaling and induction of detoxifying enzymes. Of note, nCurc was more effective than Curc in upregulating pulmonary HO-1 expression. In consistence with the results, Curc upregulated the Nrf2/HO-1 pathway and attenuated oxidative stress in lipopolysaccharide (LPS)-induced lung injury [60]. It also regulates this pathway in several other organs, either in vitro or in vivo as reviewed in [60,63,64]. Nrf2, a redox-sensitive factor, regulates cellular antioxidant responses against oxidative stress by controlling the transcription of antioxidant genes [65]. Nrf2 expression is low under basal conditions and sequestered by its repressor Keap-1. During oxidative stress conditions such as Cu toxicity, however, Nrf2 expression is strikingly elevated due to its dissociation from Keap-1. Nrf2 then migrates to the nucleus and attaches to the antioxidant response element (ARE) sequence to upregulate the expression of some genes encoding antioxidant proteins, including HO-1 [57,60]. HO-1 is responsible for heme degradation and has antioxidant, cytoprotective, and anti-inflammatory properties [31]. The anti-inflammatory properties of Curc are mediated by the activation of this enzyme via the Nrf2 and p38 MAPK signaling pathways. The capacity of Curc to reduce inflammation is abolished when HO-1 is inhibited in vascular endothelial cells [66]. In addition, Curc protects against H_2_O_2_-induced damage in lung mesenchymal stem cells via the Akt/Nrf2/HO-1 signaling pathway [67].

In light of the association between CuSO_4_ pulmonary toxicity and oxidative stress, inflammation is another logical consequence of this stress. Cu-induced lung tissue inflammation is a characteristic of augmented pathological tissue injury. Several studies reported that Cu overload induced a significant immune response and elevated IL-6 and TNF-α levels in murine bronchoalveolar lavage fluid [68] and rat liver tissue [24]. The generation of ROS by Cu is the key step behind inflammation development. Our findings are supported by the study of Kim et al. [69] who reported that excess ROS production can induce upregulation of ICAM-1 via the activation of many signaling molecules, including NF-κB. Similarly, a study by Gosens et al. showed pulmonary toxicity after inhalation of CuO NPs induced interstitial and alveolar inflammation accompanied by abundant macrophages and/or granulocytes at higher doses of CuO NPs [70]. In this regard, lung tissue inflammation was denoted by the elevated levels of pro-inflammatory cytokines, IL-6, and TNF-α and upregulation of ICAM-1 in CuSO_4_-treated rats. ICAM-1 is a cell surface glycoprotein that is normally expressed at low levels in immune, endothelial, and epithelial cells, but its expression can be upregulated by inflammatory cytokines [71]. The pro-inflammatory effects of TNF-α are exerted in part by promoting monocyte adhesion to the pulmonary epithelium and upregulation of ICAM-1 expression in an NF-κB-dependent manner [72].

On the other hand, Curc can reduce inflammation and protect the lungs from damage via other mechanisms. Curc downregulated the expression of ICAM-1 induced by NF-κB and TNF-α in lung epithelial cells [34,73,74,75]. The suppression of ICAM-1 production by Curc may have a protective effect on the integrity of the epithelial-endothelial cells by changing the interactions between their surface molecules and thereby limiting neutrophil adherence to endothelial cell monolayers in vivo [31]. Curc confers immunomodulatory actions by regulating the activation of various immune cells: T-cells, B-cells, macrophages, neutrophils, natural killer, and dendritic cells [76]. Furthermore, the anti-inflammatory activity of Curc can be partly credited to the upregulation of the Nrf2/HO-1 pathway as confirmed in Nrf2-knockout macrophages [77].

In the inflammation context, CuSO_4_ reduced the SP-C level in rat lungs, possibly due to tissue inflammation and lymphocyte infiltration. It has been reported that SP-C deficiency in human and animal models is correlated with increased inflammation and delayed healing. This protein can inhibit inflammation by decreasing JAK/STAT activation during lung repair [78]. SP-C consists of a complex mixture of phospholipids that coats the surfaces of the alveoli of the lungs where gas exchange takes place to maintain alveolar integrity and lower the surface tension that is essential for normal respiratory function. Numerous studies showed that TNF-α caused negative effects on surfactant synthesis in the lungs [79,80,81]. Furthermore, some studies revealed a strong association between surfactant C and lung diseases; for example, Stephan et al. demonstrated that the absence of SP-C or pro-SP-C is directly linked with the pathogenesis of interstitial lung disease in mice [82]. SP-C is dramatically decreased in different lung injuries and is frequently associated with apoptosis in type II alveolar epithelial cells. Inhibiting SP-C in these cells may enhance CXCL1 and 2, as well as their receptor CXCR2 and ICAM-1 expression, indicating an inflammatory response [83]. In the current study, the use of Curc or nCur almost restored the normal expression of SP-C. Guzel et al. reported that Curc significantly reduced the severity of intestinal ischemia/reperfusion injury by decreasing the activity of inducible nitric oxide synthase and increasing SP-D expression in lung tissue [84].

In addition, exposure to CuSO_4_ reduced the expression of MUC-1 in rat lungs, while Curc and nCurc restored the normal protein expression. MUC-1 is a membrane-bound glycoprotein expressed on the surfaces of all epithelial cells that line mucosal surfaces. Under physiological conditions, MUC-1 is vital in lubrication, preventing dehydration, and providing protection from degradative enzymes and microorganisms [85]. However, during exposure to pathogenic stimuli, MUC-1 exerts anti-inflammatory effects mediated by the inhibition of Toll-like receptor 5 (TLR-5) signaling [86]. MUC-1-knockout mice had more inflammation in response to flagellin, a TLR5 agonist, than wild-type mice, confirming that MUC-1 has an anti-inflammatory function during airway infection. Also, the knockdown of MUC-1 in normal human bronchial epithelial cells can induce the release of IL-8 after TLR5 agonist addition [86,87]. On the contrary, other studies reported that TNF upregulated *MUC-1* gene expression [88] or neutrophil elastase [89], suggesting that the expression of the *MUC-1* gene increased in response to inflammatory mediators to control inflammation. However, data showing the role of Cu in IL-8 and TLR expression and their association with MUC-1 are lacking; thus, future studies are needed.

Moreover, apoptosis regulatory genes were measured in order to determine the effect of CuSO_4_ in lung tissue. Cu-induced cell death might be directly related to the induced oxidative stress and inflammatory response. As expected, apoptotic cell death was found in the lungs of CuSO_4_-challenged rats, in which *Bax* and *Bcl-2* gene expression was increased and decreased, respectively. These findings align with prior research indicating that overexposure to CuSO_4_ elicits cellular apoptosis in several organs [43,44,45]. ROS and pro-inflammatory mediators induce the pro-apoptotic Bax, thus disturbing the outer mitochondrial membrane. Consequently, cytochrome c can leak out into the cytoplasm and trigger the activation of several caspases, including caspase-3, the main executioner enzyme of cell apoptosis [90]. Excessive Cu exposure induced autophagic gene expression such as *Beclin1*, reduced mitochondrial membrane potential (ΔΨm), and increased the number of dead cells as reported by TUNEL assay [91]. The anti-apoptotic BCL-2, however, inhibits the release of cytochrome c by dimerization with BAX, controlling Ca^2+^ and suppressing caspases, and thus interferes with apoptosis [42]. In agreement with previous studies [92,93], Curc showed anti-apoptotic effects in the lungs after CuSO_4_ exposure by restoring the regular expression of *BAX* and *Bcl-2* and controlling Bax/Bcl-2-mediated cell death. Nevertheless, the potency of nCurc was superior to its native form in attenuating apoptosis.

## 4. Materials and Methods

### 4.1. Chemicals

CuSO_4_, Cur, carboxymethylcellulose (CMC), trichloroacetic acid, thiobarbituric acid, reduced glutathione (GSH), pyrogallol, hematoxylin and eosin (H&E), acrylamide, agarose, and primers were procured from Sigma (St. Louis, MO, USA). Deferoxamine (DFO) and nCurc were obtained from Novartis Pharma AG (Rotkreuz, Switzerland) and Lipolife (LLT1, Essex, UK), respectively. The nCurc was encapsulated liposomal nano-curcumin to enhance the pharmacokinetic properties and systemic bioavailability by up to 98%. It was manufactured in a European laboratory registered under the Hazard Analysis Critical Control Point (HACCP) system. IL-6 and TNF-α ELISA kits were obtained from R&D Systems (Minneapolis, MN, USA), while other ELISA kits for glutathione peroxidase 2 (GPX2), ICAM-1, mucin-1 (MUC-1), and surfactant protein C (SP-C) were purchased from MyBioSource (San Diego, CA, USA). Antibodies targeting Keap1, Nrf2, HO-1, and β-actin were bought from Novus Biologicals (Centennial, CO, USA).

### 4.2. Animals and Experimental Design

A total of forty male Wistar Albino rats, weighing between 180 g and 200 g, were obtained from the Animals Research Centre at King Saud University (KSU). The experiments complied with the regulations set forth by the research ethics committee of KSU, as indicated by the ethical approval no. SE-19-129. The rats were placed in standard cages, divided into five groups with eight rats in each, and allowed to acclimate for one week. They were provided free access to water and food and kept under standard temperature, humidity, and a 12-h light/dark cycle. After one week of acclimation, the control group, designated as Group I, was given 1% C.M.C. orally. All rats in the remaining groups received 100 mg/kg CuSO_4_ [94,95], but only groups III, IV, and V were treated with daily doses of 23 mg/kg DFO [96], 80 mg/kg Curc [9,96], and 80 mg/kg nCurc [9,96], respectively. All treatments were suspended in 1% C.M.C. and administered orally for 7 days. Twenty-four hours post-treatment, the rats were sacrificed under anesthesia, blood samples were collected to obtain sera, and the lungs were harvested and rinsed with cold phosphate-buffered saline (PBS). Some tissue was snap-frozen in liquid nitrogen for RNA isolation and Western blotting experiments. The remaining tissues were preserved in 4% neutral-buffered formaldehyde for histopathological experiments and some were homogenized in Tris-HCl buffer (10% *w*/*v*, pH 7.4) and centrifuged to collect supernatants for biochemical assays.

### 4.3. Measuring Oxidative Stress Markers

The lung tissue supernatants were used to measure malondialdehyde (MDA), GSH, and SOD activity based on colorimetric methods as previously described in Ohkawa et al. [97], Ellman [98], and Marklund and Marklund [99], respectively.

### 4.4. Histological Evaluation

The fixed lung tissues were processed, embedded in paraffin wax, and then sliced into 5 µm thick sections. The sections were subsequently stained using H&E stains and visualized using light microscopy.

### 4.5. Determination of Inflammatory and Lung-Specific Biomarkers

IL-6 and TNF-α were measured using R&D Systems ELISA kits (R&D Systems, Minneapolis, MN, USA), whereas other markers, GPX2, ICAM-1, MUC-1, and SP-C, were determined using MyBioSource ELISA kits (MyBioSource, San Diego, CA, USA). All assays were carried out in compliance with the manufacturer’s protocols.

### 4.6. Gene Expression

In this study, we employed quantitative polymerase chain reaction (qPCR) to ascertain whether CuSO_4_ and treating agents changed the expression of B cell lymphoma-2 (*BCL-2*) and BCL-2-associated X protein (*BAX*). In brief, RNA extraction was performed using TRIzol reagent (Invitrogen, Waltham, MA, USA) and then the RNA concentrations were measured using Nanodrop. High-quality RNA samples were reverse-transcribed to cDNA using a high-capacity cDNA reverse transcription kit (ThermoFisher Scientific, Waltham, MA, USA). The PCR mixture contained cDNA, SYBR Green PCR master mix (ThermoFisher Scientific, Waltham, MA, USA), and the primer pairs listed in Table 1. The cycling conditions began with 10 min denaturation at 95 °C, then 40 cycles at 95 °C for 15 s, and 60 °C for 1 min. The obtained data were analyzed using the 2^−ΔΔCt^ method [100], normalized to the reference gene β-actin, and the gene expression of samples was presented as a fold change relative to controls [101].

### 4.7. Western Blotting

Changes in the protein expression of Keap1, Nrf2, HO-1, and β-actin were determined in lung homogenates in which the tissue was lysed by the addition of RIPA buffer and Protease Inhibitor CocktailX (Sigma, St. Louis, MO, USA). A Bradford protein assay kit (BioBasic, Markham, ON, Canada) was used to determine protein concentrations. A total of 50 µg of protein lysate was separated using 10% SDS-PAGE and transferred onto PVDF membranes (Millipore, Bedford, MA, USA). Following a 1 h incubation at room temperature with 5% milk, the membranes were incubated overnight at 4 °C with primary antibodies. On the next day, the membranes were thoroughly washed with TBST and then incubated with HRP-labeled secondary antibody. The signal was developed using Pierce™ ECL Western Blotting Substrate (ThermoFisher Scientific, Waltham, MA, USA), and the protein bands were acquired using ImageQuant LAS 4000 and quantified using Image J software.

### 4.8. Statistical Analysis

The data analysis and comparison of means were carried out using one-way ANOVA and Tukey’s post hoc test using GraphPad Prism 10 software (GraphPad, San Diego, CA, USA), and the data were presented as means ± standard errors of the mean (SEM). The mean differences were deemed statistically significant if the *p*-values ≤ 0.05.

## 5. Conclusions

To sum up, by addressing oxidative stress, the Nrf-2/HO-1 pathway, and inflammation in the lungs, we indicate the mechanisms underlying CuSO_4_-induced lung injury in rats and determine the efficacy of Curc and nCurc in preventing such toxicity. These beneficial effects were accompanied by the suppression of apoptotic markers and restoration of SP-C and MUC-1 expression. The enhanced Curc bioavailability in its nanoform improved lung protection against CuSO_4_. These results shed new light on Curc and nCurc and their lung-protective effects against CuSO_4_-induced lung injury, but further research is needed to investigate other associated mechanisms.

## Figures and Tables

**Table 1:** Primers used for gene expression assay.

Gene	GenBank Accession Number	Primers (5′–3′)	Amplicon Size (bp)
*BAX*	NM_017059.2	F: AGGACGCATCCACCAAGAAG R: CAGTTGAAGTTGCCGTCTGC	166
*BCL-2*	NM_016993.1	F: ACTCTTCAGGGATGGGGTGA R: TGACATCTCCCTGTTGACGC	94
*β-actin*	NM_031144.3	F: AGGAGTACGATGAGTCCGGC R: CGCAGCTCAGTAACAGTCCG	71

## Data Availability

Data analyzed or generated during this study are included in this manuscript.
